# Dataset on simulated microbond tests using finite element method: Simulation cases about different geometrical influences, material behavior, damage evolution, and element meshes

**DOI:** 10.1016/j.dib.2024.110341

**Published:** 2024-03-16

**Authors:** Royson Donate Dsouza, Donato Di Vito, Jarno Jokinen, Mikko Kanerva

**Affiliations:** a Faculty of Engineering and Natural Sciences, Tampere University of Technology, P.O. Box 589, Tampere FI-33014, Finland; b Faculty of Information Technology and Communication Sciences, Tampere University of Technology, P.O. Box 589, Tampere FI-33014, Finland

**Keywords:** Microbond, Interface, Cohesive zone model, Fracture

## Abstract

This data article provides an extensive dataset obtained from finite element (FE) simulations of microbond (MB) tests. The simulations cover a wide range of structural effects and artifacts that influence the results of the MB tests. A total of 432 simulations were performed,taking into account the various factors such as blade geometry and position, plastic behaviour of thermoset and thermoplastic droplets, material properties of the fibres, residual stresses, fracture modes at interfaces, and FE mesh sensitivity analysis. Each FE simulation consists of blade reaction force, blade displacement, fibre displacement, fibre strain and various energy metrics such as interface strain energy, total strain energy, damage energy and plastic dissipation energy. For ease of reference, the individual data files are organised in a systematic naming sequence based on the simulation matrices, detailing the specific abbreviations for each file. A user-friendly interface is also provided to read and visualisethe data from the output files in relation to the simulation matrix. For more information on the interpretation and analysis of this data, please refer to a research article entitled “Mutual dependence of experimental and data analysis features in characterization of fibre-matrix interface *via* microdroplets (R. Dsouza et al., 2023)”.

Specifications Table

**Table:** 

Subject	Engineering
Specific subject area	Computational Mechanics
Data format	Raw, Analyzed
Type of data	.txt files (dataset with numbers) .py file (Python code for user interface to analyze the data)
Data collection	The data was acquired from finite element simulations performed using Abaqus/Standard, version 2020. Necessary data is extracted from the output files of finite element simulations and stored as text files (.txt).
Data source location	• Institution: Tampere University • City/Town/Region: Tampere • Country: Finland
Data accessibility	Repository name: Mendeley Data Data identification number: 10.17632/gt2yxbt9pf.1 Direct URL to data: https://data.mendeley.com/datasets/gt2yxbt9pf/1 Instructions for accessing these data: Instructions are provided in this article
Related research article	**Authors names:** Royson Donate Dsouza, Donato Di Vito, Jarno Jokinen and Mikko Kanerva. **Title:** Mutual dependence of experimental and data analysis features in characterization of fibre-matrix interface *via* microdroplets. **Journal:** Polymer Composites **DOI:**10.1002/pc.27649 Dsouza, R., Di Vito, D., Jokinen, J., M, Kanerva., Mutual dependence of experimental and data analysis features in characterization of fiber-matrix interface *via* microdroplets, Polymer Composites, 2023.

## 1 Value of the Data

• The output data generated from the FE simulations of the MB test are computationally intensive and highly beneficial for researchers to interpret the test with various combined effects. This detailed simulation dataset fills a critical void in the current literature, which lacks comprehensive data on MB test results.
• This dataset is an excellent source to research different cohesive zone model (CZM) techniques for fibre-matrix adhesion. It enables a detailed comparison of the effects of various CZM parameters, a study not extensively available in existing research.
• The data can be used in the development of analytical models and the enhancement of experimental testing processes of the MB test.
• It provides a good foundation for data scientists to employ machine learning algorithms in correlating test data with key design parameters, such as adhesion and fatigue.

## 2 Data Description

The dataset consists output files from FE simulations, detailed in Ref. [1]. Each FE simulation results in #11 output files. To facilitate easy identification, abbreviations are used to represent the output files for specific FE models, as listed in Tables 1 and 2, along with their descriptions. Notably, energy quantities such as PD, PD_i, DMD, IE, IE_i, SE, SE_i, and ETOTAL are key indicators for understanding the material behavior in the MB test, a feature not documented elsewhere.

**Table 1:** Descriptions of abbreviations used in the simulation data.

Sl. No	Notation	Description
1	G	Glass fibre
2	CF	Carbon fibre
3	EP	Epoxy droplet
4	PP	Isotactic polypropylene droplet
5	$PP_{\Delta T}$	Isotactic polypropylene droplet with thermal load
6	$m_{1}$	Linear elastic material model
7	$m_{2}$	Elastic-perfectly plastic material model
8	$m_{3}$	Elastic-isotropic hardening material model
9	$m_{4}$	Elastic-kinematic hardening material model
10	$P_{1}$	Blade position 1
11	$P_{2}$	Blade position 2
12	$P_{3}$	Blade position 3
13	$\lambda_{1}$	Damage evolution criteria with all modes active (mode I = mode II = mode III = 130 J/m^2^)
14	$\lambda_{2}$	Damage evolution criteria with all modes active (mode I = 130 J/m^2^, mode II = mode III = 10^9^ J/m^2^)
15	$b_{p}$	Parallel blade configuration
16	$b_{c}$	Circular blade configuration
17	$\phi_{1}$	Coarse mesh FE models
18	$\phi_{2}$	Fine mesh FE models

**Table 2:** Descriptions of the abbreviations used for the output data files.

Sl.no	Notation	Description (units)
1	RF	Reaction force (N)
2	bd	Blade displacement (mm)
3	fd	Fibre displacement (mm)
4	PD	Energy dissipated by plastic deformation (mJ)
5	PD_i	Energy dissipated by plastic deformation at the interface (mJ)
6	DMD	Energy dissipated by damage (mJ)
7	IE	Total strain energy for the entire FE model (mJ)
8	IE_i	Total strain energy at the interface (mJ)
9	SE	Recoverable strain energy (mJ)
10	SE_i	Recoverable strain energy at the interface (mJ)
11	ETOTAL	Total energy for the entire model (mJ)

**Fig. 1:**
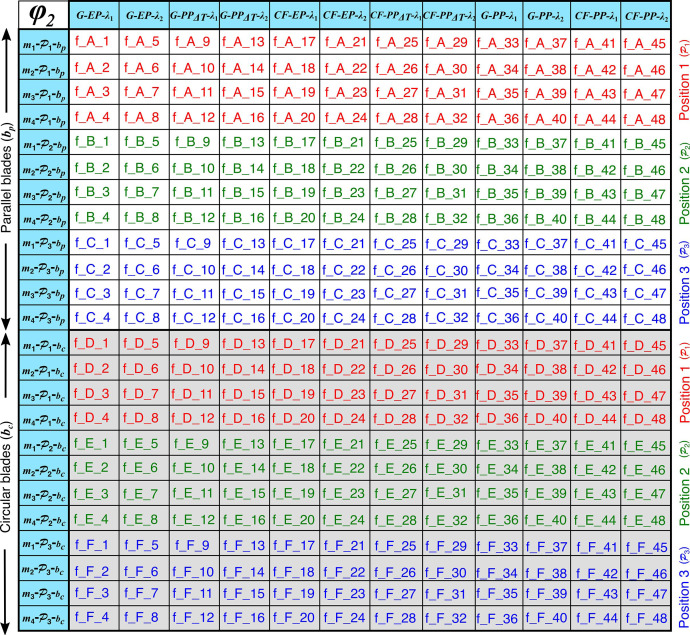
Simulation matrix for the fine mesh ($\phi_{2}$) FE models. These abbreviations are used for the output files in the folder “Data_for_DIB”.

**Fig. 2:**
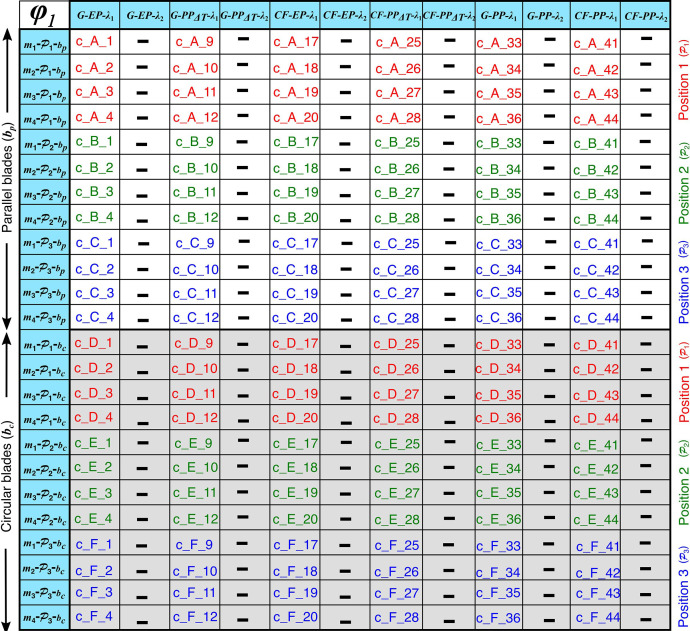
Simulation matrix for the coarse mesh (*ϕ*_1_) FE models. These abbreviations are used for the output files in the folder “Data_for_DIB”.

**Fig. 3:**
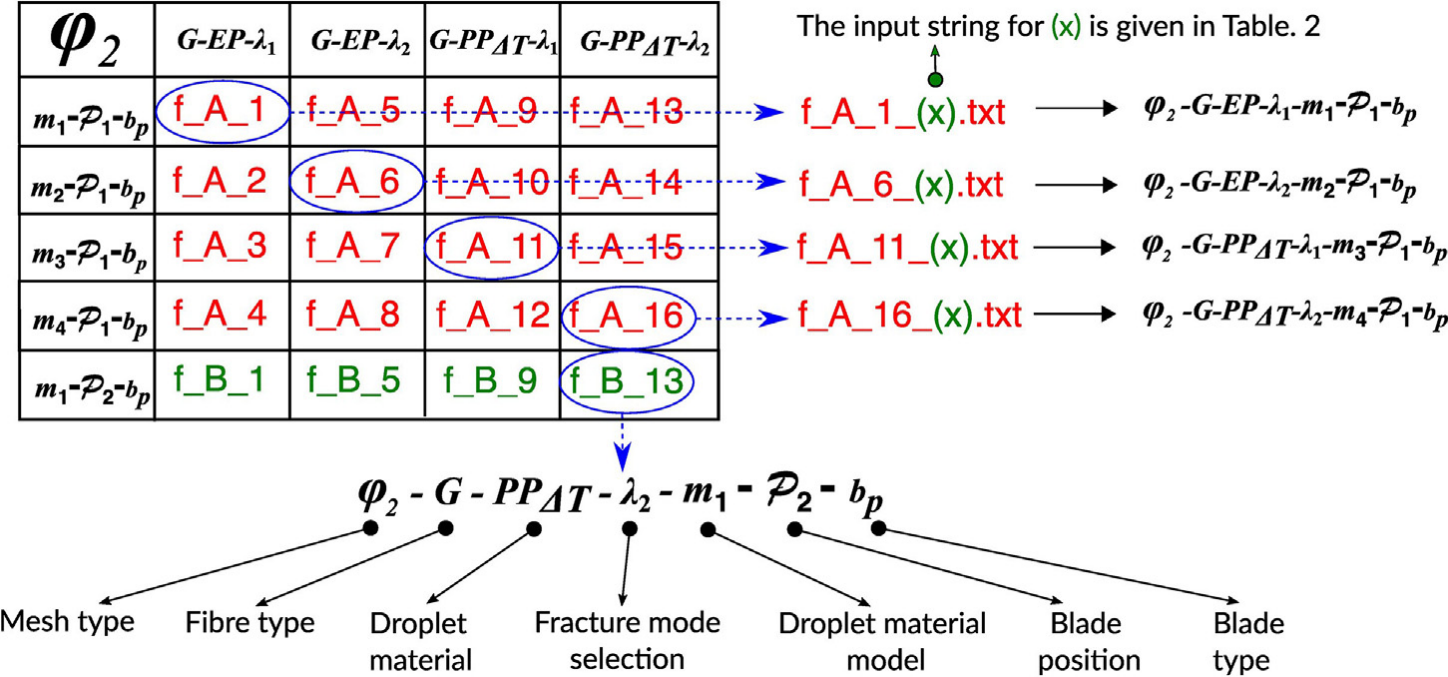
Details of individual cells in the simulation matrix.

The dataset is contained within a folder named “Data_for_DIB”. The folder includes 4752 files, each with a “.txt” extension. Users can easily locate the desired file by referring to the simulation matrix, as shown in Figs. 1 and 2. A detailed breakdown of the content in the simulation matrix is presented in Fig. 3. Two examples are shown below for clarity.

**Example 1**: If the user needs plastic dissipation energy quantity (PD) and reaction force data (RF) for:

• Standard mesh models ($\phi_{2}$).
• Glass fibre (G), epoxy droplet (EP) and damage growth criteria with all modes active ($\lambda_{1}$).
• Material model for position 1 ($P_{1}$) with parallel blades ($b_{p}$).

then, the user must refer to the first column and first row in Fig. 1 to locate the file “f_A_1”. The output .txt file will be “f_A_1_(x).txt” (see Fig. 3). Replace (x) with “PD” and “RF”, as needed. Therefore, the user-requested files are “f_A_1_PD.txt” and “f_A_1_RF.txt”. These two files are available in the “Data_for_DIB” folder.

**Example 2**: If the user needs recoverable strain energy quantity at the interface ($SE_{i}$) and blade displacement (bd) for:

• Coarse mesh models ($\phi_{1}$).
• Glass fibre (G), PP droplet with thermal load ($PP_{\Delta T}$) and damage propagation criteria with all modes active ($\lambda_{1}$).
• Material model for position 1 ($P_{1}$) with circular blades ($b_{c}$).

then, the user must refer to the first column and first row in Fig. 2 to locate the “c_D_9”. The output .txt file will be “f_D_16_(x).txt”. Replace (x) with “$SE_{i}$” and “bd” as needed. Therefore, the user-requested files are “c_D_9_SE_i.txt” and “c_D_9_bd.txt”. These two files are available in the “Data_for_DIB” folder.

Table 2 should be referred for the abbreviations of all user requested output files. It is recommended to pay close attention to the notations/abbreviations to prevent errors while using the dataset.

**Fig. 4:**
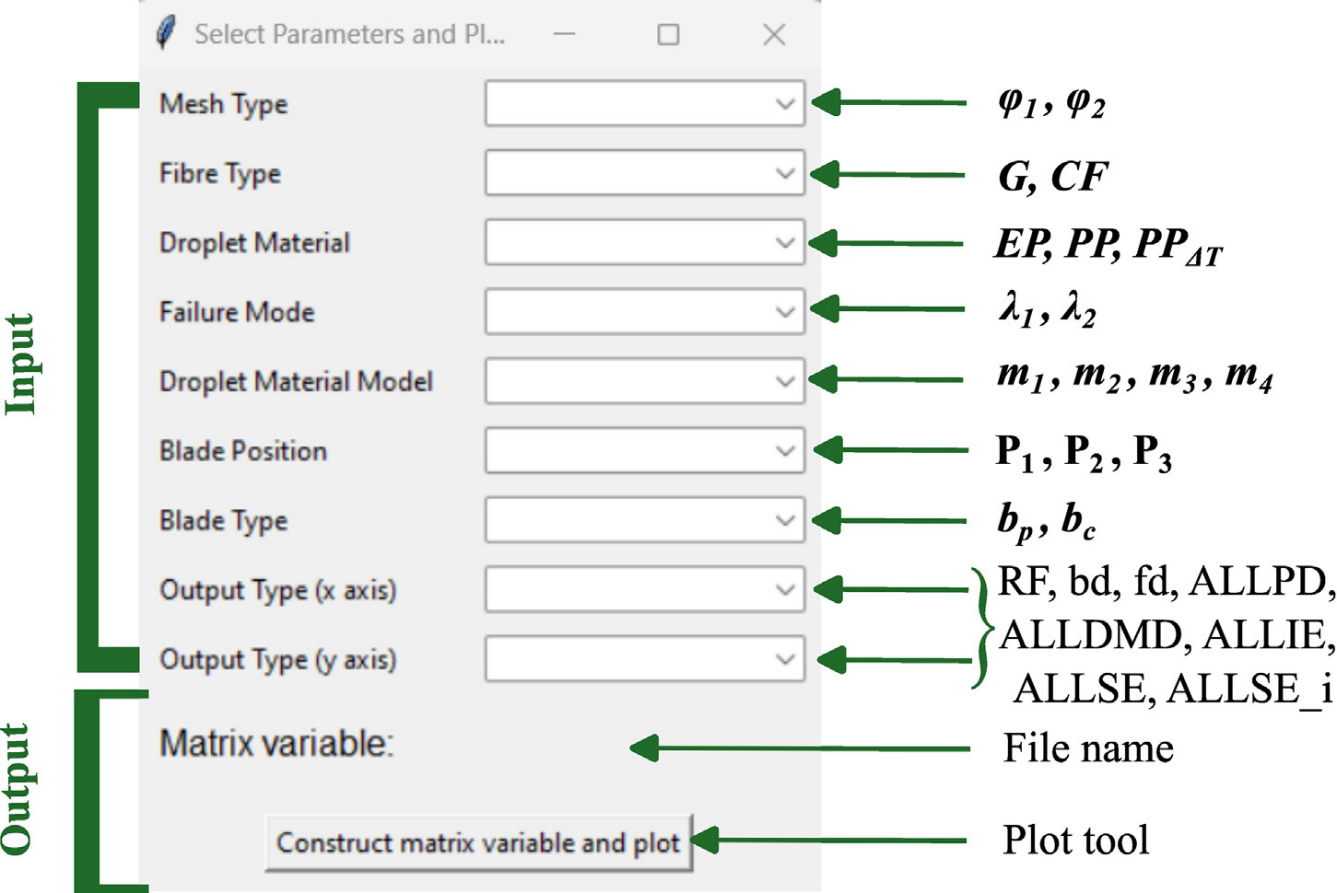
User interface options for input and output data.

**Fig. 5:**
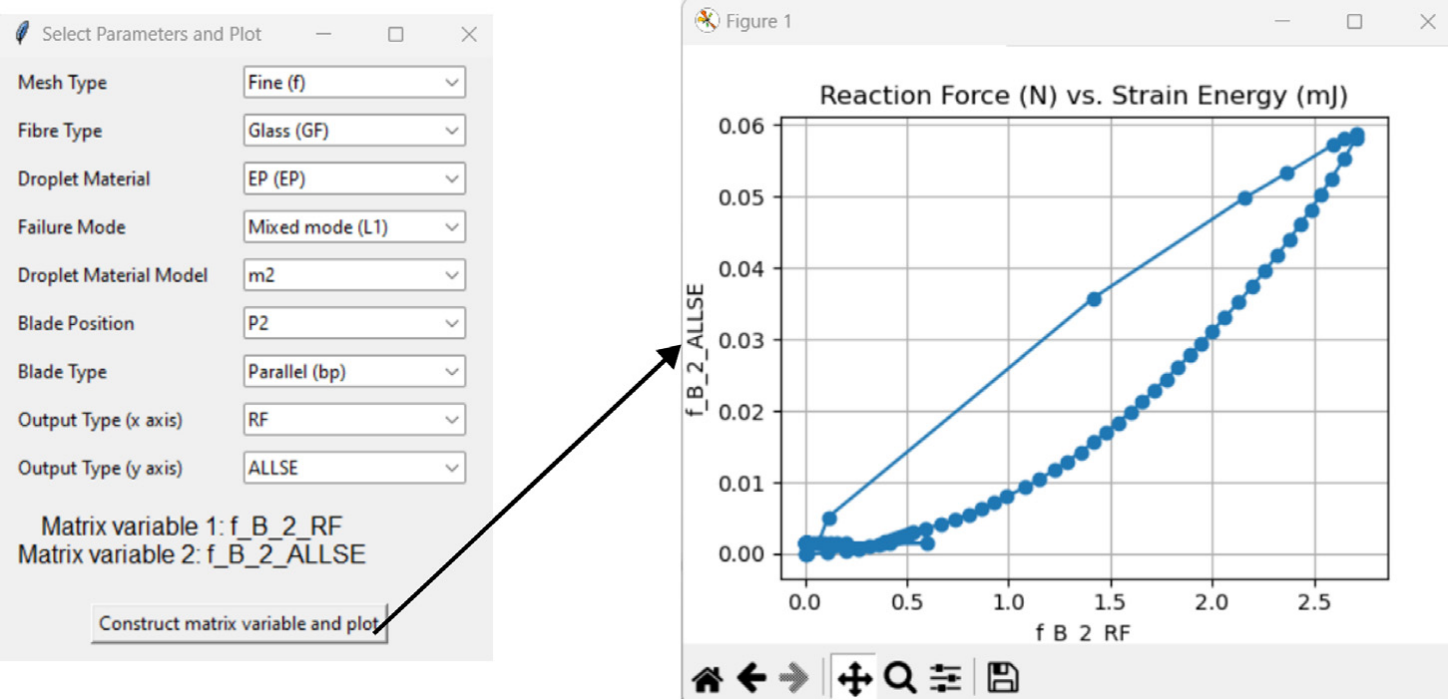
User interface after data selection and plotting.

We have developed a sophisticated yet user-friendly Graphical User Interface (GUI) leveraging the tkinter Python library (shown in Fig. 4), to facilitate the construction of matrix variables from user-selected parameters and further visualize relationships among data variables using graphical plots [2]. The GUI's feautures include parameter selection, file name construction linking simulation matrix of Figs. 1 and 2, and data visualization (Fig. 5). It supports the selection of various parameters related to mesh type, fibre type, droplet material, failure mode, droplet material model, blade position, blade type, and output types for the x and y axes. The underlying logic uses several dictionaries to map user input to specific matrix variable codes, which are then utilized to retrieve data from text files for plotting.

To use this GUI, Python and the matplotlib library must be installed, and the provided script should be stored in a directory with the necessary output files. Execute the script using the command `python interface.py´. Running this script opens the GUI window (Fig. 4), allowing users to select various parameters and visually analyze data relationships at the click of a button (Fig. 5).

## 3 Experimental Design, Materials and Methods

In general, the MB test is used to characterize the interface between the fibre and the matrix [3], [4], [5], [6]. The test samples are prepared by depositing the droplets onto the fibre followed by a curing process and subsequent cooling to room temperature. The MB test is carried out by shearing the droplet from the fibre surface using a pair of parallel blades, during which force and displacement data are recorded. Although this test has been employed for over three decades, it is subjected to structural effects and artifacts that are critical in evaluating interfacial constants.

To study various factors influencing the MB test outcomes, a comprehensive simulation matrix related to this dataset was designed. This simulation matrix considers parameters such as fibre and droplet materials, different material models for the droplet, geometrical parameters, fracture mode parameters at the interface, and the mesh effects.

The specific details along with the abbreviations used for the simulation matrix are as follows:

• Fibres: Two different types were used, namely E-glass (G) and carbon fibre (CF).
• Droplet materials: These included room-temperature-cured thermoset epoxy (EP), thermoplastic droplets such as isotactic polypropylene without the thermal load (PP) and isotactic polypropylene with the thermal load ($PP_{\Delta T}$)
• Material models for droplets: Four types were utilized, both for both thermoset and thermoplastic materials:
  ○ Linear elastic material ($m_{1}$)
  ○ Elastic-perfectly plastic ($m_{2}$)
  ○ Elastic-isotropic hardening ($m_{3}$)
  ○ Elastic-kinematic hardening ($m_{4}$)
• Blade types: Two different types were used to load the droplet, namely parallel blades ($b_{p}$) and circular blades ($b_{c}$),with three different blade positions ($P_{1}$, $P_{2}$ and $P_{3}$).
• Damage propagation criterions:Two variants were used:
  ○ With all modes active ($\lambda_{1}$)
  ○ With practically only mode I active ($\lambda_{2}$)
• Mesh types: Two different types were employed, namely coarse mesh ($\phi_{1}$) and fine mesh ($\phi_{2}$).

**Fig. 6:**
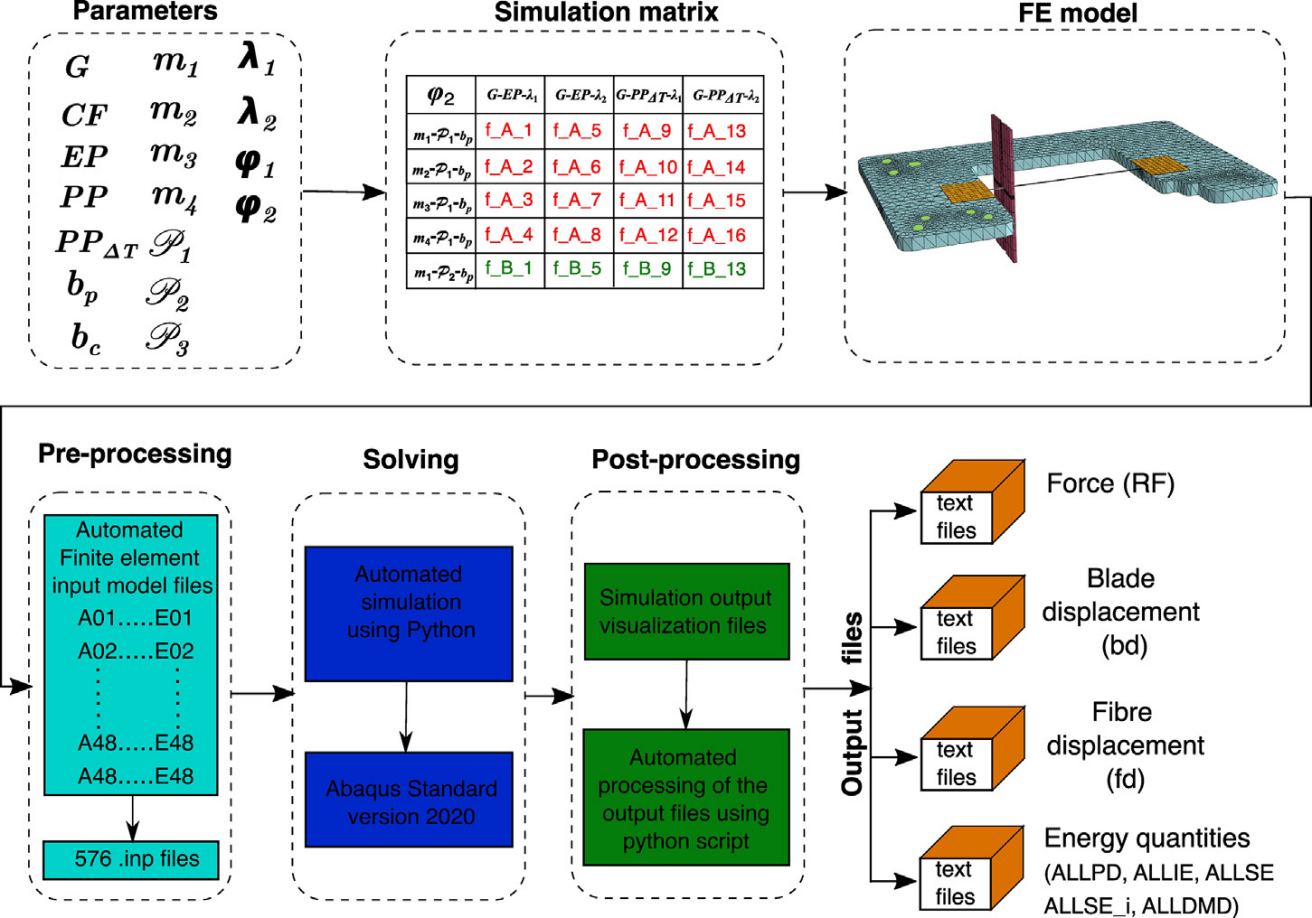
FE simulation framework along with the output data processing.

All the above parameters were used to generate a simulation matrix consisting of 432 simulation cases. While experimental datasets on the MB test are available in Ref. [10], this represents one of the most extensive datasets currently available for MB test simulations, particularly in terms of simulation files, a resource not provided by existing repositories. Every simulation case was modelled using Abaqus/Standard 2020 [7] by scripting the input files with extension “.inp” (Python scripts are available in Ref. [8]: Folder – “Input file generation”). The input files were then sequentially solved using Python code (Scripts available in Ref. [8]: Folder – “Batch running of inp files”). The Python script connected the input files to the FE solver (ABAQUS/Standard solver), generating output files with an “.odb” extension. These output database files were then processed through another python script to generate text files with specific outputs (Scripts available in Ref. [8]: Folder – “Automation for reading odb files”). Fig. 6 provides a detailed illustration of the entire FE simulation framework followed in this study. For a more comprehensive description of the simulation cases, readers are encouraged to refer to the literature [9].

## Limitations

The validity of this dataset is fundamentally linked to the accuracy of the FE model used. Limitations that inherent in the FE model, could affect the dataset's reliability for some applications. While providing in-depth insights into specific fiber-matrix adhesion scenarios, its generalizability to other fiber-matrix systems or alternative testing methods may be limited. Interpreting this dataset requires substantial expertise in FE analysis, material science, and data analysis, potentially limiting its accessibility to experts in these areas. Furthermore, the included Python scripts, referenced in Ref. [8], are designed for Python 2 and have been tested with Abaqus versions up to 2023, but not with Abaqus 2024, which requires Python 3.

## Ethics Statement

The authors have read and follow the ethical requirements for publication in Data in Brief and confirming that the current work does not involve human subjects, animal experiments, or any data collected from social media platforms.

## CRediT authorship contribution statement

**Royson Donate Dsouza:** Conceptualization, Methodology, Software, Investigation, Writing – original draft. **Donato Di Vito:** Software, Formal analysis. **Jarno Jokinen:** Writing – review & editing. **Mikko Kanerva:** Conceptualization, Writing – review & editing, Supervision, Funding acquisition.

## Data Availability

Finite Element Analysis Dataset: Microbond Tests with Diverse Cases and Parameters (Original data) (Mendeley Data).
